# Quality of care: assessment

**DOI:** 10.1186/1476-511X-6-12

**Published:** 2007-04-19

**Authors:** Allam Appa Rao, Gumpeny Ramachandra Sridhar

**Affiliations:** 1 Department of Computer Sciences and Systems Engineering, Andhra University, Visakhapatnam, India; 2 Endocrine and Diabetes Centre, Visakhapatnam, India

## Abstract

To translate science into clinical practice we must first assess the quality of care that is being delivered. The resulting information about qualitative and quantitative parameters can then be assessed. Ultimately insights can be obtained into improving the quality of care in diabetes mellitus. The Diabetes Quality Improvement Programme in USA has shown such an exercise is feasible. A similar exercise in India is necessary to improve the quality of diabetes care.

## Background

Complications in diabetes can be reduced by good glycemic control [1,2], and by correction of coexisting abnormalities such as dyslipidemia and hypertension [3]. In addition, lifestyle factors such as lack of physical exercise and smoking are potentially correctable. They are common in persons with diabetes who present for medical attention [4]. The bottleneck in diabetes care is not lack of evidence, but difficulty in implementing what is already known [5].

It is necessary to assess the quality of care that is being delivered so that it serves as a benchmark for further improvement.

## Domains of quality

Quality can be assessed on the following areas (a) Well being, quality of life [6], (b) Clinical and biochemical parameters (c) Economic aspects [7].

All three components are important in chronic lifestyle diseases such as diabetes mellitus, where we aim at care rather than cure [6]. One must carefully consider a combination of all three aspects and must not aim for improvement in one area, to the exclusion of others.

## Assessment of quality

Guidelines must be available to assess the quality of care in diabetes. The Diabetes Quality Improvement Programme (DQIP) [8] identified 'standardized' uniform performance measures for diabetes care, following a national consensus on care assessment.

At the outset a distinction must be made between performance measures and care guidelines. Performance measures assess level of care delivered *across an entire population*, whereas care guidelines recommend desired level of care *for a single patient*.

The aim and requirements of the DQIP were to develop a measurement set that is both accepted and implementable. Necessarily that required organizational, financial and logistical support.

The operating group was broad based and included nearly all of the organizations concerned with delivery of diabetes care, including but not limited to American Diabetes Association, American Society of Internal Medicine, American College of Physicians and Centre for Disease Control and Prevention.

Similarly the technical expert panel comprised a broad section of professionals: endocrinologists, internists, family physicians, dieticians, educators, nurses, epidemiologists, and experts in performance measurement. The last was critical in providing expertise in an area where clinicians are not generally competent. When such a study is carried out in India, the experience derived from the DQIP can serve as a framework.

The accountability measures had to be comprehensive yet parsimonious to minimize the burden of data collection. If they were so elaborate to collect information, the very purpose is lost. Similarly the data must be sufficient to draw valid conclusions. Extensive pre release evaluation was carried out in the development stages. Similarly data collection feasibility studies were also done, using paper forms and electronic format. Meticulous planning and execution led to the success of the programme.

Ultimately what contributed to its success were: (a) belief that opportunities exist to reduce burden of diabetes, (b) assessment of quality of care improves diabetes care (c) centralized performance measures gives 'collective power'. The motto was to ultimately *translate science into clinical practice*.

Following the DQIP, sub studies were done, such as SCRIPT: Study of Clinically Relevant Indicators of Pharmacologic Therapy. Ultimately DQIP could form a template for performance assessment. An assessment was made of the percentage of patients with diabetes between 18 and 75 who had each of the following investigations or results during a one year period: HbA1c tested at least once, poor HbA1c level (ie high), ocular fundus examined, lipid profile done, lipid control (LDL<130 mg/dl), monitor for nephropathy, blood pressure control, foot exam done.

Doctors had flow sheets and reference sheets. Patients and families had access to brochures, health records and reminder cards.

MedQuest software contains software for data entry system design and for data collection. MedQuest software components included those for *Data entry *to capture data, *Analyzer *to build variables, clinical indicators, manager for system environment, quality performance in patients, lexicon database and an ICD9 database.

## Application to clinical care

Outcomes can be predicted on the basis of biographical and clinical variables. For examples a recent study has shown that the following characteristics were predictive of standard care: those aged 18–24 years, more clinic visits, use of insulin, and area with more beds [9]. The measures are applicable and practical. However it is necessary that the information must be utilized to improve quality of care [10]. Mere documentation is a job only half done.

Similarly psychological variables predicted better quality of life. Subjects with strong belief in self-efficacy and an optimistic outlook had better satisfaction and quality of life [11].

Patient education must be comprehensive and include: information about diabetes and its treatment, nutritional management, physical activity, medications, glycemic monitoring, prevention, detection and treatment of complications, and finally psychosocial adjustment [12].

Evaluating compliance might uncover indicators such as cultural background and ethnicity [13]. Or physical barriers can be identified such as transportation, cost and commitment [14]. It is also possible that some of the observations may suggest cost-effective delivery of health care by primary care physicians rather than sub-specialist endocrinologists or diabetologists: a multi centric study of processes and outcomes among groups of specialist physicians and generalists showed that there was no significant difference in outcomes with specialist physicians, other than better patient satisfaction [15].

## Applicability to developing countries

Assessing quality of care is comparable to applied research. Evidence is available that metabolic and glycemic control prevent and postpone complications. We must put the evidence to practice. In view of Indian diabetes centres with large computerized databases, baseline data analysis is possible. This provides information of age structure, sex, socio economic status, follow up, compliance, as well as prevalence of cost-effective modifiable factors.

As the next step a consensus can be reached on quality measures, which can be followed by networking different centres, either as paper or electronic records [16,17]. While electronic networking is not yet widely available the advantages of electronic records are obvious [18]. Connectivity can be applied to local area networking among different work areas in one Centre, as in the MVDSC Chennai model [17] or may be extended over a wider geographical area, though limited to fewer clinical care areas.

Besides the use in quality assessment, and improvement, clinical database networking offers many other advantages such as improved pattern recognition, interaction with patients, tailored health information to patients, and secondary evidence based database development [18].

Published literature has shown that in busy primary care practices electronic medical records and computerized tracking systems are necessary to sustain improved diabetes care [19]. A companion presentation on electronic management system (EMS) concluded that EMS improved performance outcomes, but not metabolic outcomes [20]. Therefore longer study involving new care delivery models may be needed to improve metabolic outcomes (ie better glycemic control, lower blood pressure, correction of dyslipidemia), along with performance outcomes (ie, carrying out the tests).

A variety of data mining methods can be employed to extract information data that would be generated [21-23]. These may provide novel associations that are useful to both clinicians and administrators. The goal is to make available medical knowledge more functional.

A beginning has been made in an international study, the Diabetes Care Excellence Project (DCEP) involving seven clinics, including MV Diabetes Specialities Center in India [21]. The purpose of the study is to 'document standard definition used at different centers on an international basis, and to assess the feasibility of collection and comparison of data on quality of care in diabetes in real-life settings.'

## Conclusion

Given the availability of robust evidence-based data for achieving metabolic control, it is necessary to translate principles to practice. Putting in place quality improvement systems is a crucial part of this translation.

## Competing interests

The author(s) declare that they have no competing interests.

## Authors' contributions

*AAA *and *GRS *participated in designing and writing of the manuscript. Both authors have read and approved the final manuscript.
